# Effects of short-term ingestion of Russian Tarragon prior to creatine monohydrate supplementation on whole body and muscle creatine retention: a preliminary investigation

**DOI:** 10.1186/1550-2783-9-S1-P24

**Published:** 2012-11-19

**Authors:** Jonathan M Oliver, AR Jagim, A Sanchez, K Kelley, Elfego Galvan, James Fluckey, S Riechman, Mike Greenwood, Ralf Jäger, M Purpura, I Pischel, Richard B Kreider

**Affiliations:** 1 Department of Health and Kinesiology, Exercise and Sport Nutrition Laboratory, Texas A&M University, College Station, TX 77843, USA; 2 Increnovo LLC, 2138 E Lafayette Pl, Milwaukee, WI, 53202, USA; 3 PhytoLab GmbH & Co. KG, Dutendorfer Straße 5-7, 91487 Vestenbergsgreuth, Germany

## Background

It has been well-established that creatine monohydrate (CrM) increases whole body creatine retention and muscle creatine content. Extracts of Russian Tarragon (RT) have been reported to produce anti-hyperglycemic effects [1] and influence plasma creatine levels during the ingestion of CrM [2]. Theoretically, RT ingestion with CrM may promote greater creatine retention than ingesting CrM alone. The purpose of this preliminary study was to determine if short-term, low-dose aqueous RT extract ingestion prior to CrM supplementation influences whole body creatine retention or muscle creatine content.

## Methods

In a double-blind, randomized, and crossover manner; 10 untrained males (20±2 yrs; 179±9 cm; 91.3±34 kg) ingested 500 mg of aqueous Tarragon extract (*Finzelberg*, *Andernach*, *Germany*) or 500 mg of a placebo (P) 30-minutes prior to ingesting 5 g of CrM (*Creapure^®^*, *AlzChem AG*, *Germany*) (CrM+RT). Subjects ingested the supplements two times per day (morning and evening) for 5-days and then repeated the experiment after a 6-week wash-out period. Urine was collected at baseline and during each of the 5-days of supplementation to determine urine creatine content. Whole body creatine retention was estimated as the difference from orally ingested CrM (10 g/d) from the amount of creatine excreted daily in urine. Muscle biopsies were also obtained from the *vastus lateralis* at baseline and after 3 and 5 days of supplementation for determination of muscle free creatine content. Data were analysed by MANOVA with repeated measures.

## Results

Daily urinary excretion of creatine increased in both groups from baseline (0.4±0.5; 1.9±1.4, 3.5±2.4, 4.4±3.2, 3.9±2.6, 5.2±3.1 g/d; p=0.001) with no differences observed between groups (CrM+P 0.34±0.4, 1.9±1.6, 3.5±2.3, 4.7±3.3, 3.2±2.8, 5.0±3.4; CrM+RT 0.5±0.6, 1.7±1.1, 3.4±2.7, 4.2±3.3, 4.6±2.2, 5.4±3/2 g/d; p=0.59). Whole body daily creatine retention increased following supplementation (0.0±0.0; 8.2±1.4, 6.5±2.4, 5.6±3.2, 6.1±2.6, 4.8±3.2 g/d; p=0.001) with no differences observed between groups (CrM+P 0.0±0.0, 8.1±1.6, 6.5±2.4, 5.3±3.2, 6.8±2.8, 5.0±3.4; CrM+RT 0.0±0.0, 8.3±1.1, 6.6±2.7, 5.8±3.3, 5.4±2.2, 4.6±3.2 g/d; p=0.59). Total whole body creatine retention during the supplementation period were not significantly different among groups expressed in total grams retained (CrM+P 31.7±11.1; CrM+RT 30.6±10.3 g; p=0.82) or percentage retained (CrM+P 63.4±22.3%; CrM+RT 61.2±19.9%; p=0.82) over the supplementation period. There was significant variability in muscle phosphagen levels, therefore, only muscle free creatine data are reported. After 3 and 5-days of supplementation, respectively, both supplementation protocols demonstrated a significant increase in muscle free creatine content from baseline (4.8±16.7, 15.5±23.6 mmol/kg DW, p=0.01) with no significant differences observed between groups (CrM+P 9.3±14.3, 22.8±28.2; CrM+RT 0.3±18.4, 8.1±16.2 mmol/kg DW; p=0.34). In percentage terms, muscle free creatine content in both groups increased over time (p=0.008) by 10.9±27% and 23.5±34% after 3 and 5-days, respectively, with no differences observed between groups (CrM+P 0.0±0.0, 21.1±30, 37.3±42; CrM+RT 0.0±0.0, 0.7±21, 9.6±18 %, p=0.13).

## Conclusions

Results indicate that ingesting as little as 5g of CrM taken twice daily increases total muscle creatine content by 23.5±34.5%. However, our preliminary findings indicate that ingesting RT 30-min prior to CrM supplementation did not affect whole body creatine retention or muscle free creatine content during a short-period of creatine supplementation (10 g/d for 5-days) in comparison to ingesting a placebo prior to CrM supplementation. Additional research is needed with a larger sample size to examine: 1.) whether ingestion of greater amounts of RT prior to and/or in conjunction with CrM ingestion would affect creatine retention; 2.) whether ingestion of RT with CrM over longer periods of time would affect creatine retention; and, 3.) whether co-ingesting RT with CrM and carbohydrate may reduce the need for ingesting carbohydrate with CrM in order to promote greater creatine retention.
